# The cerebrovascular response to graded Valsalva maneuvers while standing

**DOI:** 10.1002/phy2.233

**Published:** 2014-02-10

**Authors:** Blake G. Perry, Toby Mündel, Darryl J. Cochrane, James D. Cotter, Samuel J. E. Lucas

**Affiliations:** 1 School of Sport and Exercise, Massey University, Palmerston North, New Zealand; 2 School of Physical Education, Sport and Exercise Sciences, University of Otago, Dunedin, New Zealand; 3 Department of Physiology, University of Otago, Dunedin, New Zealand; 4 School of Sport, Exercise and Rehabilitation Sciences, College of Life and Environmental Sciences, University of Birmingham, Birmingham, United Kingdom

**Keywords:** Cerebral blood flow, orthostasis, syncope, Valsalva maneuver

## Abstract

The Valsalva maneuver (VM) produces large and abrupt increases in mean arterial pressure (MAP) at the onset of strain (Phase I), however, hypotension, sufficient to induce syncope, occurs upon VM release (phase III). We examined the effect of VM intensity and duration on middle cerebral artery blood velocity (MCAv) responses. Healthy men (*n *=**10; mean ± SD: 26 ± 4 years) completed 30%, 60%, and 90% of their maximal VM mouth pressure, for 5 and 10 sec (order randomized) while standing. Beat‐to‐beat MCAv and MAP during phase I (peak), at nadir (phase III), and recovery are reported as the change from standing baseline. During phase I, MCAv rose 15 ± 6 cm·s^−1^ (*P *<**0.001), which was not reliably different between intensities (*P *=**0.11), despite graded increases in MAP (*P *<**0.001; e.g., +12 ± 9 mmHg vs. +35 ± 14 for 5 sec 30% and 90% VM, respectively). During Phase III, the MCAv response was duration‐ (*P* = 0.045) and intensity dependent (*P *< 0.001), with the largest decrease observed following the 90% VM (e.g., −19 ± 13 and −15 ± 11 cm·s^−1^ for 5 and 10 sec VM, respectively) with a concomitant decrease in MAP (*P *<**0.001, −23 ± 11 and −23 ± 9 mmHg). This asymmetric response may be attributable to the differential modulators of MCAv throughout the VM. The mechanical effects of the elevated intrathoracic pressure during phase I may restrain increases in cerebral perfusion via related increases in intracranial pressure; however, during phase III the decrease in MCAv arises from an abrupt hypotension, the extent of which is dependent upon both the duration and intensity of the VM.

## Abstract (precis)

More intense Valsalva maneuvers when standing are associated with an increase blood pressure response during Phase I of the maneuver although this is not accompanied by changes in cerebral blood flow. However, following the maneuver (phase III) more intense straining is associated with a greater decrease in both blood pressure and cerebral blood flow and in some instances is sufficient to induce syncope.

## Introduction

The Valsalva maneuver (VM) is defined as a forced exhalation against a closed glottis (Hamilton et al. 1936) and is executed during coughing (Hamilton et al. 1944), defecation, and also during resistance exercise (MacDougall et al. 1992). The VM can be separated into four distinct phases: Phase I, an increase in mean arterial pressure (MAP) at the onset of the strain as the elevated intrathoracic pressure is translated to the arterial circulation; phase IIa, a reduction in stroke volume as atrial filling pressure is reduced; phase IIb, an increase in heart rate mediated by the arterial baroreflex to offset the reduction in stroke volume; phase III, a rapid decline in MAP as the strain is released; phase IV, rapid recovery and overshoot of MAP as the now restored cardiac output is ejected into a constricted arterial tree (Goldberg et al. 1952; Tiecks et al. 1995; Pott et al. 2000).

The VM may be viewed as eliciting undesirable cardiovascular and cerebrovascular responses, but there is also evidence that it may indeed protect the cerebral circulation during phase I of the maneuver (Tiecks et al. 1995; Niewiadomski et al. 2012). Specifically, increases in intrathoracic pressure are translated to the cerebrospinal fluid (Hamilton et al. 1944) such that increases in intracranial pressure (ICP) ensue (Greenfield et al. 1984), reducing transmural pressure in the cerebral arteries (Haykowsky et al. 2003). This alteration in transmural pressure may mechanically restrain the passive dilation of vessels downstream of the middle cerebral artery (MCA) in response to the greatly elevated perfusion pressure. Via a maintained resistance, flow would be limited and would culminate in no change in middle cerebral artery blood velocity (MCAv). Furthermore, the increased central venous pressure (CVP) experienced during a VM may reduce the pressure difference across the cerebral vascular bed (Pott et al. 2000). Nevertheless, while these mechanical mechanisms may limit the increase in MCAv, an elevation is observed during phase I (Tiecks et al. 1995) and this reflects the high‐pass filter characteristics of the cerebral circulation (Zhang et al. 1998).

Many studies investigating the hemodynamic response to the VM have adopted a supine position, which alleviates the orthostatic component and consequently reduces the phase III response (Pott et al. 2000). Pott et al. (2000) examined the effect of a moderate intensity VM on the hemodynamic response while standing, however, this was submaximal (40 mmHg mouth pressure) and of 15‐sec duration, which may not necessarily reflect many conditions where a VM is recruited in everyday life. Tiecks et al. (1995) assessed the effects of VM intensity (20 and 40 mmHg mouth pressure) on the cerebrovascular response, reporting no difference in the phase III decrease between these pressures. However, these VMs were all performed while supine and, therefore, did not include the orthostatic component that can influence this response (Pott et al. 2000). Interestingly, a vigorous VM while sitting upright has been reported to induce syncope (Duvoisin 1961), and of course syncope is apparent during orthostatic stress (Julu et al. 2003). As the VM is commonly recruited in the standing position during resistance exercise, everyday tasks (lifting) and is a similar maneuver to coughing, documentation of the hemodynamic responses at different intensities while standing would be advantageous for the identification of the risk factors that may induce syncope in otherwise healthy humans during normal everyday tasks. Thus, the purpose of this investigation was to examine the hemodynamic response to graded VMs while standing. The working hypothesis was that the VM will protect the brain against hyperperfusion during phase I of the maneuver; that is, the increase in MAP during phase I will be intensity‐dependent, however, no change in MCAv will be observed. In addition, following the release of the VM (phase III), an increased VM intensity will induce a greater hypotension and be matched by an intensity‐dependent concomitant decrease in MCAv.

## Methods

Ten healthy nonsmoking men were recruited for the study (mean ± SD: age, 26 ± 4 years; body mass, 93 ± 12 kg; height, 181 ± 8 cm). All participants were resistance trained and had a training age of 4.5 ± 3.0 years. Participants were informed of the potential risks and experimental procedures, and informed written consent was obtained. All procedures and protocols were approved by the University of Otago Human Ethics Committee and performed in accordance with the *Declaration of Helsinki*. All participants were free from disease and were not taking any medication. Participants abstained from strenuous exercise, alcohol, and caffeine for at least 24 h before the experimental trial.

### Study design

Participants visited the laboratory on two occasions: once during familiarization and once during the experimental trial. During the familiarization session the participants were familiarized with all experimental procedures and equipment, including practicing VMs at end‐inspiration following a quiet period of spontaneous breathing. This enabled pre‐VM hyperventilation to be minimized during experimental trials. Mouth pressure served as a surrogate for intrathoracic pressure (MacDougall et al. 1985; Morgan et al. 1993; Convertino et al. 2003; Heffernan et al. 2007) and reportedly reflects changes in oesophageal pressure (Goldberg et al. 1952; Flemale et al. 1988). All VMs were performed in the standing position.

### Experimental protocol

During the experimental trial each participant would first stand for 5 min during which baseline measures were obtained. Participants upon instruction would then complete a maximal VM for 10 sec. Following recovery (i.e., when all values returned to baseline), relative VMs of 30%, 60%, and 90% of the maximal Valsalva pressure were then performed for both 5 and 10 sec, the order of which (both intensity and duration) was randomized. Visual feedback of the absolute mouth pressure was given in real time in order to aid the participant. Each VM was separated by 5 min or until values have returned to baseline. Participants were verbally instructed what pressure and duration to obtain immediately before the performance.

### Measurements

Blood flow velocity in the right MCAv was measured using a 2‐MHz pulsed Doppler ultrasound system (DWL; Compumedics Ltd, Singen, Germany) using search techniques described elsewhere (Aaslid et al. 1982; Willie et al. 2011). The Doppler probe was secured with a plastic headband (DWL) to maintain the insonation angle throughout the protocol. Participants breathed through an adjustable mouthpiece, which allowed for the measurement of mouth pressure and the partial pressure of end‐tidal CO_2_ (P_ET_CO_2_; gas analyzer model ML206; ADInstruments, Sydney, Australia). Mouth pressure was measured via a transducer attached to the mouthpiece and was used to measure the pressure during all VMs. Blood pressure was measured noninvasively and continuously using finger photoplethysmography (Finapres Medical Systems, Biomedical Instruments, Amsterdam, The Netherlands), and heart rate was measured via three‐lead electrocardiogram (ADInstruments). Finger blood pressure values were checked against a manual sphygmomanometer initially and regularly during rest periods. If the two were not in agreement the finger cuff was replaced and/or the hand was warmed until the pressures matched. The hand was kept at heart level throughout each trial. All data were acquired continuously via an analogue‐to‐digital converter (PowerLab ML870; ADInstruments) at 1 kHz. Data were displayed in real time and recorded for off‐line analysis using commercially available software (v7.3.3 Lab Chart; ADInstruments).

Mean blood flow velocity (MCAv_mean_) and mean arterial blood pressure (MAP) were calculated as the integral for each cardiac cycle divided by the corresponding pulse interval. Cerebral vascular conductance (CVC) was calculated via the equation MCAv_mean_/MAP. Relative to baseline measures, the percentage decrease in MCAv_mean_ was divided by the percentage reduction in MAP at MCAv_mean_ nadir to assess differences in the MAP contribution to the reduction in MCAv_mean_. The Gosling pulsatility index for MCAv was calculated as MCAv_systolic_ − MCAv_diastolic_/MCAv_mean_ (Gosling and King 1974).

### Data analyses

Baseline data were acquired in the last minute of each baseline period between VMs, and presented as the mean across that minute. All variables at the attainment of the “peak” for both the MCAv_mean_ and MAP phase I responses were used in the analysis (i.e., a data point for each individual peak). Time to peak was taken from the start of the VM to the MCAv_mean_ and MAP peaks independently. Each VM was analyzed for MCAv_mean_ and MAP times and magnitude of nadir (phase III), and time to recovery from the end of the strain (similar to Stolz et al. 2010). Nadir was defined as the lowest measured value immediately following the VM, and recovery as the point when the variable was equal to the baseline value during the ascent from nadir. Additionally, the area under the curve (AUC) from phase IIa to recovery (ascending from the phase III nadir) was calculated (Pruessner et al. 2003).

Inferential statistical analyses of dependent variables were performed using two separate three‐way analyses of variance (ANOVAs) (intensity × duration × phase): (1) change from baseline, and; (2) time to peak (during phase I), nadir (Phase III), and recovery (early phase IV). Intensity refers to the relative intensity of the VM performed and duration the length the strain was held for (either 5 or 10 sec). Phase refers to the comparison of a specific time point to the baseline reference value (i.e., peak and nadir). The AUC was analyzed within each duration using a one‐way ANOVA. Data were assessed for approximation to a normal distribution and sphericity, with no corrections required. Main effects were isolated using post‐hoc pairwise comparisons (Bonferroni corrected, where necessary). All data were analyzed using SPSS statistical software (v20; Chicago, IL), with a priori statistical significance set at *P *≤**0.05. All data are presented as the mean ± SD absolute and/or relative change from the baseline preceding the VM.

## Results

**Figure 1.**
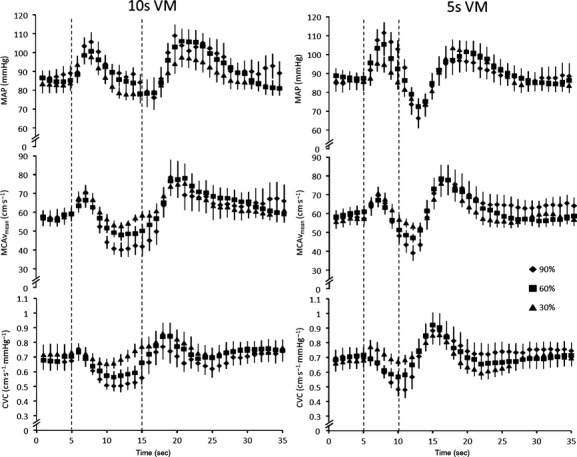
The response of mean middle cerebral artery blood flow velocity (MCAv_mean_), mean arterial pressure (MAP) and cerebrovascular conductance (CVC) during and following a Valsalva maneuver (VM) at 30%, 60%, and 90% of maximal VM pressure, displayed every second. The vertical dashed lines represent the initiation and completion of the VM. All values are means ± SE.

**Figure 2.**
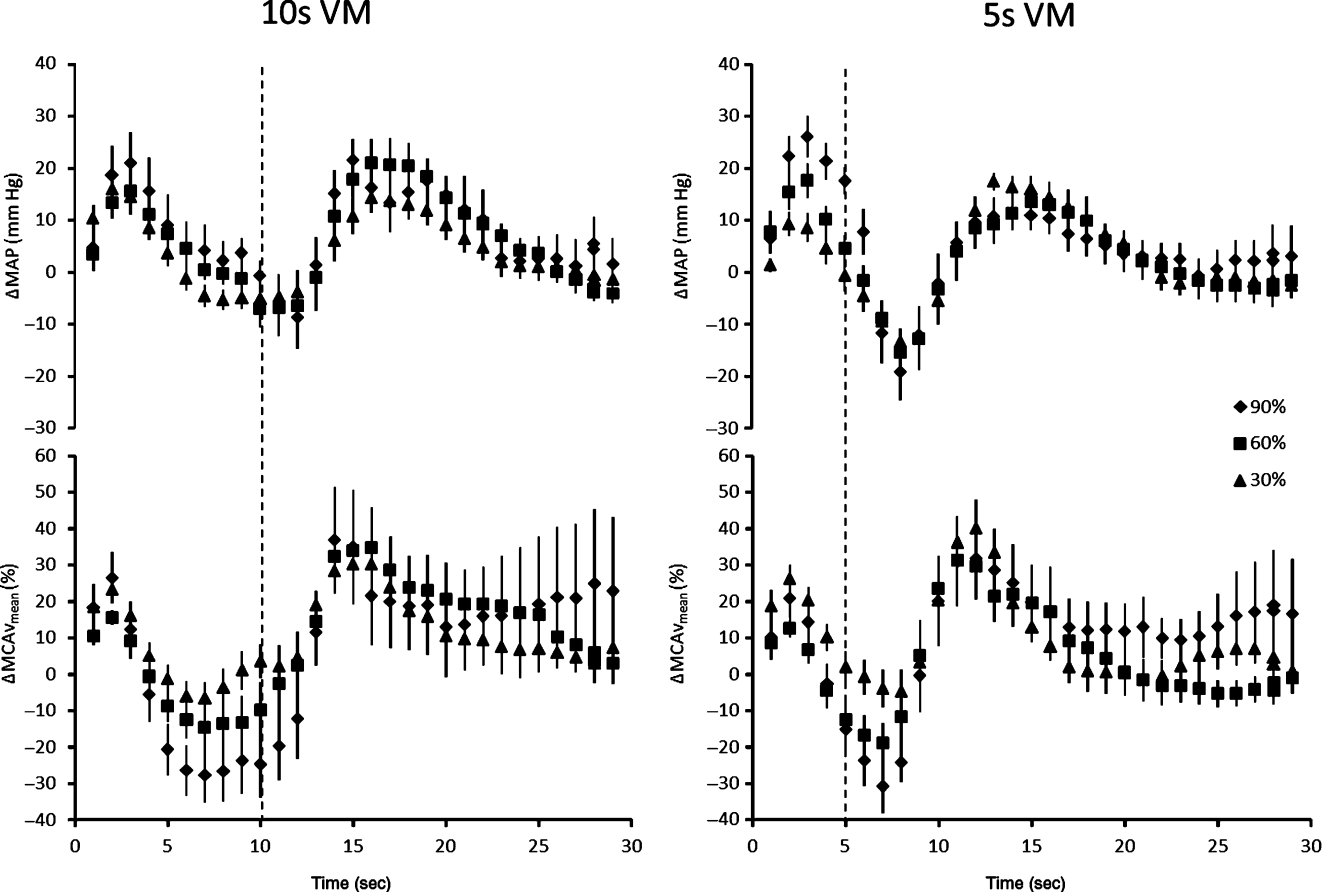
The percentage change from baseline for mean middle cerebral artery blood flow velocity (MCAv_mean_) and the absolute change in mean arterial pressure (MAP) during and following a Valsalva maneuver (VM) at 30%, 60%, and 90% of maximal VM pressure, displayed every second. The zero time point represents the initiation of the VM with the vertical dashed lines representing the completion. All values are means ± SE.

Absolute and relative changes from baseline during the VM (phase I) are displayed in Table 1 and also in Figures 1, 2. Briefly, despite MAP increasing with VM intensity, systolic, diastolic, and MCAv_mean_ remained unchanged (see Table 1 for *P* values). Changes from baseline following the VM at the nadir in phase III are shown in Table 2 and also in Figures 1, 2. In contrast to Phase I of the VM, once the strain was released larger reductions in MAP were associated with greater reductions in systolic, diastolic, and MCAv_mean_ flow velocities (see Table 2 for *P* values). In support of this, the AUC analysis for MCAv indicated a significant effect of intensity for both 10 (*P *=**0.002, −6 ± 58, −51 ± 92, −114 ± 129 aU for 30%, 60%, and 90% intensities, respectively) and 5‐sec VM (*P *=**0.004, 3 ± 36, −38 ± 43, −48 ± 62 aU for 30%, 60%, and 90% intensities, respectively). This cerebral hypoperfusion was sufficient to produce syncope in two participants but only following the maximal and 90% 10‐sec VMs. Interestingly, similar reductions in MAP during phase III were observed for both the 5 and 10 sec 90% VM, however, MCAv_mean_ was lower following the 5‐sec VM (Table 2). The pulsatility index was significantly different between VMs driven by the intensity‐by‐phase interaction (*P *=**0.002). The greatest change between the phase I and III responses was observed during the 90% VM with 0.74 ± 0.27 and 0.74 ± 0.15 during phase I for 10 and 5 sec, respectively, versus 1.29 ± 0.34 and 1.52 ± 0.61 during phase III. Baseline data including P_ET_CO_2_ (grouped mean 32 ± 5 mmHg) were unchanged between baseline periods. These data were then grouped, the means of which are displayed in Tables 1, 2 (all *P *>**0.05). Mouth pressures of 22 ± 6, 45 ± 12, and 67 ± 19 mmHg were measured during 30%, 60%, and 90% VM intensities, respectively.

**Table 1.** Changes from baseline at peak (phase I) for 30%, 60%, and 90% VM intensities.

Variable	Baseline	Time (sec)	Δ From baseline			*P* values						
			30	60	90	Intensity	Duration	Phase	I × D	I × P	P × D	I × D × P
MCAv_mean_ (cm/sec)	57 ± 9	5	15 ± 7 (27 ± 12)	14 ± 5 (23 ± 6)	16 ± 8 (29 ± 13)	0.11	0.78	<0.0001	0.63	0.11	0.78	0.62
		10	12 ± 5 (22 ± 9)	14 ± 5 (24 ± 7)	17 ± 9 (32 ± 13)							
Systolic MCAv (cm/sec)	95 ± 15	5	11 ± 9 (12 ± 9)	12 ± 6 (12 ± 6)	12 ± 12 (13 ± 13)	0.24	0.87	<0.0001	0.50	0.24	0.87	0.50
		10	10 ± 8 (12 ± 9)	10 ± 6 (11 ± 6)	16 ± 7 (17 ± 8)							
Diastolic MCAv, (cm/sec)	40 ± 6	5	15 ± 7 (38 ± 18)	13 ± 5 (31 ± 10)	16 ± 8 (40 ± 20)	0.41	0.50	<0.0001	0.85	0.51	0.50	0.85
		10	13 ± 6 (32 ± 13)	13 ± 6 (32 ± 14)	16 ± 9 (41 ± 25)							
CVC (cm·sec^−1^·mmHg^−1^)	0.69 ± 0.16	5	0.050 ± 0.12 (10 ± 16)	−0.050 ± 0.079 (−6 ± 11)	−0.11 ± 0.11 (−15 ± 15)*	0.002	0.79	0.25	0.037	0.002	0.79	0.037
		10	−0.0040 ± 0.063 (1 ± 11)	−0.027 ± 0.12 (−3 ± 17)	−0.058 ± 0.12 (−6 ± 17)*							
MAP (mmHg)	85 ± 13	5	12 ± 9 (14 ± 10)	24 ± 14 (29 ± 17)*	35 ± 14 (41 ± 19)*^,^*	<0.001	0.96	<0.001	0.23	<0.001	0.96	0.23
		10	17 ± 9 (21 ± 11)	21 ± 14 (25 ± 16)*	30 ± 22 (36 ± 24)*^,^*							
Systolic BP (mmHg)	131 ± 17	5	13 ± 11 (10 ± 8)	31 ± 22 (24 ± 18)	38 ± 19 (30 ± 16)*	0.020	0.60	<0.001	0.05	0.020	0.60	0.046
		10	29 ± 19 (23 ± 14)	28 ± 25 (21 ± 19)	35 ± 27 (27 ± 19)*							
Diastolic BP (mmHg)	70 ± 12	5	11 ± 8 (16 ± 12)	22 ± 15 (31 ± 19)*	32 ± 15 (49 ± 26)*	<0.001	0.77	0.29	0.29	<0.001	0.77	0.29
		10	14 ± 7 (22 ± 11)	19 ± 13 (27 ± 18)*	28 ± 21 (41 ± 28)*							
HR **(**bpm)	80 ± 10	5	6 ± 12 (7 ± 15)	7 ± 11 (10 ± 15)	11 ± 11 (14 ± 13)	0.083	0.90	0.021	0.66	0.083	0.90	0.66
		10	4 ± 14 (6 ± 19)	10 ± 9 (13 ± 13)	11 ± 17 (14 ± 22)							

Values are absolute mean ± SD change from baseline, with percentage change shown in parentheses. VM, Valsalva manoeuvre; MCAv, middle cerebral artery velocity; CVC, cerebrovascular conductance; MAP, mean arterial pressure; BP, blood pressure; HR, heart rate; interactions are shown for intensity (I), duration (D), and phase (P).

* Statistically different from 30%, *P *≤**0.05.

* Trend for a difference between 90% and 60%, *P *=**0.07.

Time to peak MCAv_mean_ (pooled mean 0.7 ± 0.5 sec) and peak MAP (pooled mean 1.2 ± 0.6 sec) during phase I of the VM was unaffected by Valsalva pressure (*P *=**0.40 and 0.50, respectively) or duration (*P *=**0.69 and 0.74, respectively). However, time to peak MCAv_mean_ was significantly shorter (*P = *0.004) than time to peak MAP. Time to nadir following the VM for MCAv_mean_ (pooled mean 0.5 ± 0.5 sec) and MAP (pooled mean 0.8 ± 0.4 sec) showed no significant difference between intensities (*P *=**0.40 and 0.39, respectively) or durations (*P *=**0.58 and 0.47, respectively). However, similar to the time to peak responses, the nadir occurred earlier for MCAv_mean_ than MAP (*P *=**0.017). Finally, time to recovery following the VM showed no effect of VM intensity (*P *=**0.30 and 0.62) or duration (*P *=**0.73 and 0.33, respectively) for MCAv_mean_ (pooled mean 1.6 ± 1.0 sec) or MAP (pooled mean 3.6 ± 4.3 sec); however, MCAv_mean_ recovered before MAP (*P *=**0.006).

**Table 2.** Changes from baseline at Nadir (phase III) for 30%, 60%, and 90% VM intensities.

Variable	Baseline	Time (sec)	Δ From baseline			*P* values						
			30	60	90	Intensity	Duration	Phase	I × D	I × P	P × D	I × D × P
MCAv_mean_ (cm/sec)	57 ± 9	5	−6 ± 9 (−9 ± 15)	−16 ± 11 (−25 ± 18)*	−19 ± 13 (−31 ± 22)*^,^*	<0.001	0.045	0.002	0.90	<0.001	0.045	0.90
		10	−3 ± 7 (−5 ± 13)	−11 ± 12 (−19 ± 19)*	−15 ± 11 (−26 ± 20)*^,^*							
Systolic MCAv (cm/sec)	95 ± 15	5	−9 ± 14 (−8 ± 14)	−15 ± 19 (−14 ± 20)	−24 ± 16 (24 ± 14)*	0.004	0.84	0.004	0.83	0.004	0.84	0.83
		10	−10 ± 10 (−10 ± 10)	−18 ± 20 (−17 ± 20)	−22 ± 18 (−22 ± 19)*							
Diastolic MCAv (cm/sec)	40 ± 6	5	−5 ± 11 (−9 ± 26)	−17 ± 13 (−38 ± 29)	−20 ± 15 (−47 ± 35)*^,^*	<0.001	0.077	0.004	0.40	<0.001	0.077	0.40
		10	−1 ± 9 (−4 ± 21)	−9 ± 13 (22 ± 32)	−18 ± 12 (−44 ± 28)*^,^*							
CVC (cm·sec^−1^·mmHg^−1^)	0.69 ± 0.16	5	0.081 ± 0.11 (15 ± 19)	0.026 ± 0.12 (3 ± 18)	0.044 ± 0.15 (7 ± 22)	0.58	0.33	0.017	0.76	0.58	0.33	0.76
		10	0.085 ± 0.097 (10 ± 12)	0.059 ± 0.16 (6 ± 22)	0.10 ± 0.055 (15 ± 10)							
MAP (mmHg)	85 ± 13	5	−16 ± 7 (−19 ± 8)	−18 ± 16 (−21 ± 20)	−23 ± 11 (−28 ± 13)*	0.006	0.21	0.70	0.70	0.006	0.21	0.70
		10	−12 ± 7 (−14 ± 9)	−14 ± 12 (−18 ± 16)	−23 ± 9 (−28 ± 11)*							
Systolic BP (mmHg)	131 ± 17	5	−17 ± 16 (−19 ± 10)	−24 ± 29 (−19 ± 22)	−40 ± 14 (−32 ± 11)*	0.003	0.29	0.001	0.84	0.003	0.29	0.84
		10	−25 ± 14 (−14 ± 13)	−23 ± 29 (−18 ± 19)	−37 ± 24 (−29 ± 17)*							
Diastolic BP (mmHg)	70 ± 12	5	−13 ± 7 (−18 ± 10)	−14 ± 12 (−20 ± 19)	−17 ± 10 (−25 ± 14)	0.040	0.31	0.65	0.65	0.040	0.31	0.65
		10	−10 ± 7 (−14 ± 10)	−11 ± 10 (−17 ± 14)	−17 ± 6 (−26 ± 9)							
HR **(**bpm)	80 ± 10	5	13 ± 7 (17 ± 10)	15 ± 11 (19 ± 14)*	22 ± 14 (28 ± 18)*	<0.001	0.038	<0.001	0.015	0.038	0.038	0.015
		10	14 ± 8 (17 ± 10)	24 ± 9 (31 ± 13)*^,^*	35 ± 13 (43 ± 17)*^,^*							

Values are absolute mean ± SD change from baseline, with percentage change shown in parentheses. VM, Valsalva manoeuvre; MCAv, middle cerebral artery velocity; CVC, cerebrovascular conductance; MAP, mean arterial pressure; BP, blood pressure; HR, heart rate. Interactions are shown for intensity (I), duration (D), and phase (P).

* Trend for a difference between 60% and 30%, *P *=**0.084.

* Statistically (*P *≤**0.05) different from 30%.

* Statistically different from 60%.

* Statistically different from 5s, *P *≤**0.05.

## Discussion

The main novel findings of this study were that: (1) Across the range of intensity and duration of VM we tested, increases in MCAv during phase I were comparable despite the progressive increase in MAP with VM intensity; (2) higher VM intensities resulted in a greater reduction in both MCAv and MAP upon release of the VM (phase III), and (3) time to peak, nadir, and recovery occurred earlier for MCAv than MAP. Consistent with our hypothesis, we observed an asymmetrical response in cerebral blood flow control during rapid increases and decreases in perfusion pressure. Specifically, while the MAP response to VM was found to be intensity‐dependent for both phases I and III, the MCAv response did not match this during phase I but did during phase III. These findings illustrate how the VM both challenges and contributes to the regulation of cerebral blood flow. The following discussion outlines the evidence, some implications, and methodological considerations supporting this conclusion.

### The hemodynamic response during phase I of the VM

The rapid time course of the blood pressure response during a VM (particularly Phase I) occurs too quickly (~1 sec) to be counteracted by cerebral myogenic autoregulatory processes (Greenfield et al. 1984). Therefore, one might expect that the greater increase in MAP associated with a more intense VM would cause a proportionately larger increase in MCAv. While there was an increase in MCAv during phase I relative to baseline, consistent with the high‐pass filter characteristics of the cerebral circulation (Zhang et al. 1998), it was not reliably different between intensities despite the prevailing MAP response. In addition, when we included the maximum VM data in the analysis and compared it with the 30%, 60%, and 90% 10‐sec VMs (using a one‐way ANOVA), the VM intensity still had no distinguishable effect on MCAv_mean_ (*P *=**0.09), yet MAP was further elevated.

The maintenance of MCAv despite the greater MAP response (Table 1) may be attributable to the mechanical effects of the increased intrathoracic pressure during a VM. The intrathoracic pressure is rapidly translated to the cerebrospinal fluid at the onset of the VM such that ICP rises (Hamilton et al. 1944; Greenfield et al. 1984) and reduces the transmural pressure within the cerebral arteries (Haykowsky et al. 2003). The reduction in transmural pressure may restrain the passive dilation in response to the acute increases in cerebral perfusion pressure during phase I of the VM and subsequently increases in MCAv are restrained. Furthermore, right atrial pressure increases linearly with expiratory pressure (Korner et al. 1976) and may attenuate the pressure difference across the cerebral circulation. However, blood flow velocity in the straight sinus has been reported to increase during phase I, although this may be due to the partial collapse of walls of the sinus due to an increased ICP, and therefore increased velocity may be due to changes in vessel diameter rather than flow per se (Stolz et al. 2010). This is further complicated in the standing position as the jugular vein is collapsed (Dawson et al. 2004; Gisolf et al. 2005), which may increase cerebral venous resistance (thus acting as a Starling resistor) and subsequently venous flow is redirected through the vertebral venous plexus (Gisolf et al. 2004).

**Figure 3.**
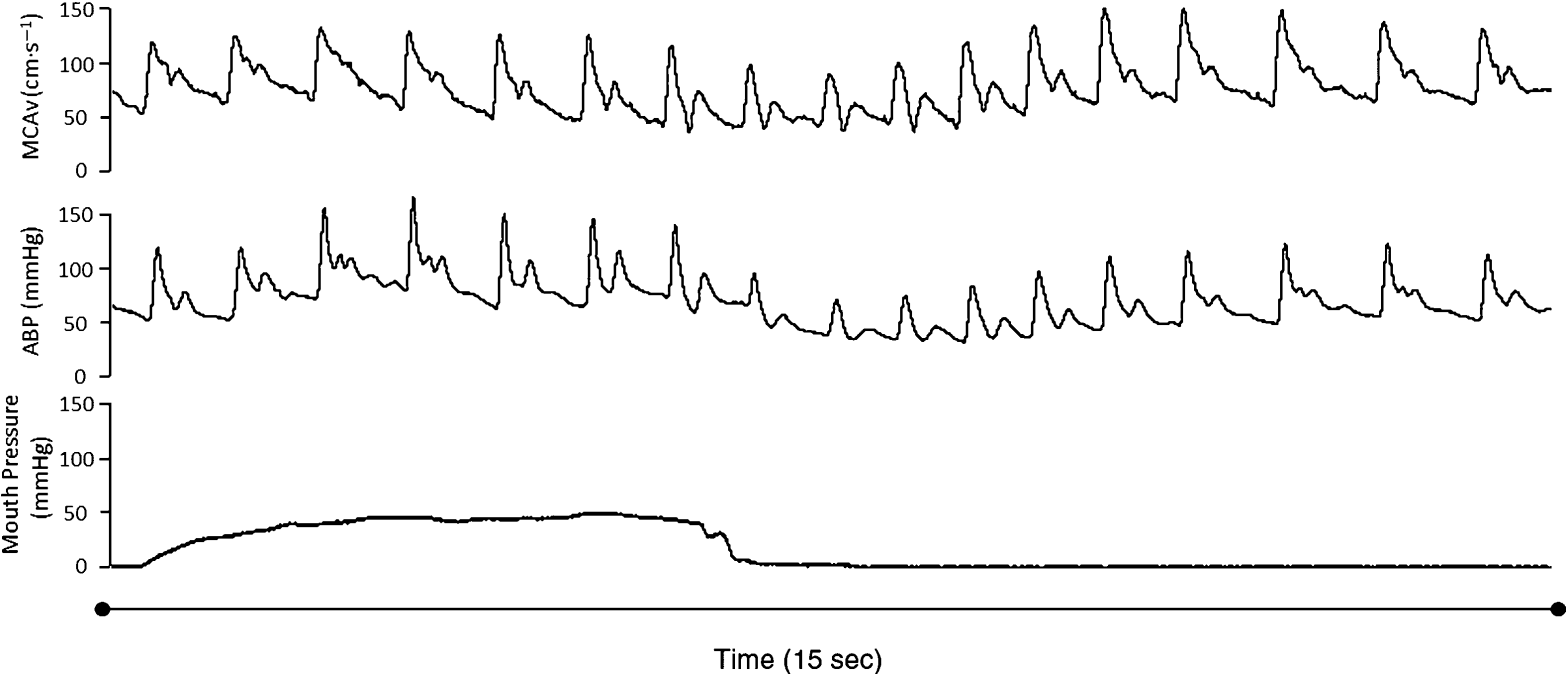
Representative trace of middle cerebral artery blood flow velocity (MCAv), arterial blood pressure (ABP) and mouth pressure during a 90% 5 sec VM in one participant.

While an autoregulatory myogenic response cannot be excluded during phase I, the rapid transfer of intrathoracic pressure to the cerebrospinal fluid (Hamilton et al. 1944) likely occurs before dynamic autoregulation has fully counteracted the increase in MAP. Dynamic cerebral autoregulation has an inherent latency of ~5 sec (Zhang et al. 1998) and our observed peak MCAv was achieved 0.7 sec following the onset of the VM. Therefore, a likely explanation for the restrained MCAv (i.e., despite the increasing MAP with greater VM intensity) would seem to be a graded resistance due to VM intensity‐dependent rises in ICP and possibly CVP, protecting the brain from possible hyperperfusion injury. This may also explain the differences in pulsatility observed between the Phase I and III responses, where during phase I a mechanical restraint of dilation (elevated ICP) would reduce the pulsatility index and alter the waveform between the phases (Fig. 3). The increase in pulsatility may maintain cerebral blood flow (CBF) via pulsatile flow when perfusion pressures are low, as observed during phase III of the VM. This may be a compensatory mechanisms that promotes flow at the lower limits of cerebral autoregulation (Lewis et al. 1999). Furthermore, the effect of this resistance to flow is apparent when comparing the hemodynamic response between phase I and the overshoot during phase IV. Despite similar increases in MAP during phase I and IV, the MCAv increase is greater during phase IV (Tiecks et al. 1995) where ICP would be expected to be declining to near baseline levels (Greenfield et al. 1984). Other mechanisms of regulation, such as the autonomic nervous system, may operate during these latter phases (phase IV) of the VM to regulate cerebral blood flow (Zhang et al. 2004). Also during squat stand maneuvers, where changes in MAP occur without changes in ICP, high frequency (0.1 Hz) changes in MCAv occur concomitantly with MAP (Claassen et al. 2009). Thus, given our MAP and MCAv responses during Phase I of the VM were not matched, it appears that the difference may be explained by changes in ICP restraining MCAv.

### The hemodynamic response during phase III of the VM

Unlike during phase I of the VM, the reduction in MAP and MCAv in phase III was dependent on VM intensity, such that the more intense VMs produced a greater reduction in both MAP and subsequently MCAv (Table 2). This was further evidenced by the occurrence of syncope in two participants while performing their maximal and 90% (10 sec) VMs, with another three participants showing symptoms of pre‐syncope (light‐headedness). This rapid reduction in MAP is likely attributable to the passive effect of intrathoracic pressure on the arteries (Tiecks et al. 1995; Dawson et al. 1999) and the refilling of the distended pulmonary vessels (Pott et al. 2000). Similar to phase I of the VM, the cerebral autoregulation myogenic response is too slow to counteract this acute hypotension, such that MCAv matches the drop in MAP, at least initially (Fig. 1). Given that the nadir of MCAv occurred before that of MAP, it would appear that the dynamic myogenic response was functional and played a role in limiting the hypoperfusion of the brain.

To the best of the authors' knowledge this is the first study to investigate the duration of the VM on the hemodynamic response. Interestingly, MCAv appears to fall more following the 5‐sec than 10‐sec VM during phase III (Table 2). This may be attributable to the release of the maneuver coinciding with the reduction in cardiac output during phase IIa (Pott et al. 2000) (no plateau in MAP trace, Fig. 1) and subsequently a larger decrease in MAP (Table 2). Although cerebral blood flow and cardiac output are causally linked (Ogoh et al. 2005), it is unlikely that a simple relation exists during the VM (Pott et al. 2003). Higher heart rates following the 10‐sec VM indicate a baroreflex‐mediated contribution to the circulatory stability (Table 2), presumably in an attempt to offset the reduction in stroke volume (Pott et al. 2000); interestingly this effect was not apparent in the shorter 5 sec VMs.

### The effect of posture on the VM

The phase III response is exacerbated in the standing position, as the reduction in MCAv during this phase is very minor while supine (Pott et al. 2000). Furthermore, Tiecks et al. (1995) found that graded VMs do not produce a greater hypotension during phase III while semi‐recumbent with no associated change in MCAv from baseline (Tiecks et al. 1995). In the same study MCAv was increased from baseline during phase I of the VM while supine but unchanged between graded pressures of 20 and 40 mmHg (mouth pressure), which is consistent with our results and a ICP‐mediating role for CBF regulation. Therefore, the hemodynamic response during phase III of the VM appears highly posture‐dependent whereas the phase I appears to be unaltered. When standing, the severe and rapid reduction in MAP during phase III ultimately challenges cerebral oxygenation sufficiently to induce syncope (Van Lieshout et al. 2003).

This study utilized healthy resistance trained individuals who commonly perform VMs in the standing position. Moreover, these individuals were free from disease and the results indicate operative cerebral regulatory mechanisms. Resistance trained individuals have a reduced central arterial compliance, which includes the carotid artery (Bertovic et al. 1999; Miyachi et al. 2004). Whether this alteration in compliance extends to the MCA is unknown. Thus, it is possible that sedentary young individuals and endurance athletes, who have an increased arterial compliance (Vaitkevicius et al. 1993), may have an altered response to the VM. Given the popularity and therapeutic effect of resistance exercise, diseased individuals with impaired regulatory mechanisms of both systemic and cerebrovascular circulations may participate in upright exercises in which the VM may be inadvertently recruited. Moreover, the VM is also recruited in everyday tasks (defecation, lifting). Thus, further research is required to identify the hemodynamic response and associated risks (and/or benefits) of performing a VM when standing in compromised and sedentary individuals and also endurance athletes.

### Technological considerations

We used transcranial Doppler ultrasound as a surrogate for cerebral blood flow, which provides a measure of blood flow velocity rather than absolute flow. The change in flow velocity has been found to accurately reflect changes in absolute flow as long as conduit artery diameter is unchanged (Valdueza et al. 1997). Importantly, the MCA diameter has been reported to change <4% during changes in MAP similar to those which we observed here (30 ± 16 mmHg) (Giller et al. 1993). The possibility of sympathetic activation cannot be excluded, especially during phase IV of the VM (Zhang et al. 2004). Furthermore, the retest reliability has been shown to be strong during repeated VMs using transcranial Doppler (Wallasch and Kropp 2012). We attempted to measure carotid artery diameter in order to clarify the effect of the VM on conduit artery diameter. However, due to the large increases in CVP, jugular pressure rises and causes the carotid to shift from the initial position. Acquisition of an adequate image within the VM time frame was not possible and thus it was not possible for data to be recorded. Therefore, the exact response of the conduit arteries, including the MCA, to the VM is unknown.

Alterations in arterial CO_2_ alter the efficacy of cerebral autoregulation (Aaslid et al. 1989). The measurement of P_ET_CO_2_ served as a substitute for arterial PCO_2_. We observed that P_ET_CO_2_ was unchanged between baselines and thus cerebral tone would have been similar at the onset of the VM. The time course of the vascular response to changes in arterial CO_2_ is asymmetric with the “on” constant much slower than the “off” (Poulin et al. 1996). The time constant of the increase in MCAv during a step change in P_ET_CO_2_ is ~6 sec (Poulin et al. 1996, 1998). Pott et al. (2000) reported that the reduction in arterial PCO_2_ contributed to 10–15% of the reduction in MCAv during a 15‐sec VM. As the maximal duration of the VM in this experiment was 10 sec the influence of changes in arterial PCO_2_ would be expected to be less, although the exact effect of possible changes in arterial CO_2_ tension during the VM performed here is unknown. However, due to the delay in the vascular response and moderate changes in arterial PCO_2_ reported during longer VMs (Pott et al. 2000), the main driving factor during and initially following the VM appears to be the rapid changes in perfusion pressure.

## Conclusion

The MAP response to phase I of the VM was intensity‐dependent, while the MCAv response was similar across the range of intensities we tested. At the end of the straining, the reduction in both MAP and MCAv was intensity dependent, resulting in marked transient reductions in MCAv, which were sufficient to induce syncope in some instances. These results were consistent with our hypothesis, and indicate that the VM may protect the brain from hypertension at the onset the VM, presumably as a result mostly of the mechanical effects of the elevated intrathoracic pressure. The time to peak (phase I) and nadir (phase III) for MCAv occurred before those of MAP. Therefore, the regulation of cerebral perfusion during the distinct phases of the VM are likely different and are not simply pressure passive.

## Acknowledgments

The authors thank the participants.

## Conflict of Interest

None declared.
